# Autoantibodies to Harmonin and Villin Are Diagnostic Markers in Children with IPEX Syndrome

**DOI:** 10.1371/journal.pone.0078664

**Published:** 2013-11-08

**Authors:** Vito Lampasona, Laura Passerini, Federica Barzaghi, Carlo Lombardoni, Elena Bazzigaluppi, Cristina Brigatti, Rosa Bacchetta, Emanuele Bosi

**Affiliations:** 1 Center for Translational Genomics and Bioinformatics, San Raffaele Hospital Scientific Institute, Milan, Italy; 2 Telethon Institute for Gene Therapy, Division of Regenerative Medicine, Stem Cells and Gene Therapy, San Raffaele Hospital Scientific Institute, Milan, Italy; 3 Diagnostica e Ricerca San Raffaele, Milan, Italy; 4 Diabetes Research Institute, San Raffaele Hospital Scientific Institute, Milan, Italy; 5 Vita Salute San Raffaele University, Milan, Italy; The University of Texas Medical Schoo at Houstonl, United States of America

## Abstract

Autoantibodies to enterocyte antigens harmonin (75 kDa USH1C protein) and villin (actin-binding 95 kDa protein) are associated with the Immune dysregulation, Polyendocrinopathy, Enteropathy, X-linked (IPEX) syndrome. In this study we evaluated the diagnostic value of harmonin and villin autoantibodies in IPEX and IPEX-like syndromes. Harmonin and villin autoantibodies were measured by a novel Luminescent-Immuno-Precipitation-System (LIPS) quantitative assay, in patients with IPEX, IPEX-like syndrome, Primary Immunodeficiencies (PID) with enteropathy, all diagnosed by sequencing of the *FOXP3* gene, and in type 1 diabetes (T1D), celiac disease and healthy blood donors as control groups. Harmonin and villin autoantibodies were detected in 12 (92%) and 6 (46%) of 13 IPEX patients, and in none of the IPEX-like, PID, T1D, celiac patients, respectively. All IPEX patients, including one case with late and atypical clinical presentation, had either harmonin and/or villin autoantibodies and tested positive for enterocyte antibodies by indirect immunofluorescence. When measured in IPEX patients in remission after immunosuppressive therapy or hematopoietic stem cell transplantation, harmonin and villin autoantibodies became undetectable or persisted at low titers in all cases but one in whom harmonin autoantibodies remained constantly high. In one patient, a peak of harmonin antibodies paralleled a relapse phase of enteropathy. Our study demonstrates that harmonin and villin autoantibodies, measured by LIPS, are sensitive and specific markers of IPEX, differentiate IPEX, including atypical cases, from other early childhood disorders associated with enteropathy, and are useful for screening and clinical monitoring of affected children.

## Introduction

Immune dysregulation, Polyendocrinopathy, Enteropathy, X-linked (IPEX) syndrome is a monogenic autoimmune disease characterized by severe enteropathy, type 1 diabetes (T1D) and eczema [1], [2]. The syndrome is caused by mutations in the *FOXP3* gene, responsible for severe impairment of regulatory T (Treg) cells [3]. While the genetic analysis is the elective method for the ultimate diagnosis, there is no clear genotype-phenotype correlation and the disease course varies among different patients. In addition, despite IPEX classification as an immunodeficiency, there are no clear immunological parameters predictors of disease severity or responsiveness to therapy [4]–[6]. Furthermore, disorders with a similar clinical phenotype, referred to as IPEX-like syndromes, may exist in the absence of *FOXP3* mutations, posing difficulties for the clinical management and therapeutic choices [4]–[6]. Therefore, the identification of markers specifically associated with the immune dysfunction of IPEX would be extremely helpful for diagnostic purposes. Circulating enterocyte autoantibodies, detected by indirect immunofluorescence, were described in the past in association with a variety of enteropathies, including those eventually identified as IPEX syndrome [7], but the molecular targets of these serological markers have long been unknown. A distinct enterocyte autoantigen recognized by sera of IPEX patients was then identified as the 75 kDa AIE-75 protein [8], [9], and further characterized as the Usher Syndrome I C (USH1C) protein, also known as harmonin [10], a scaffold protein reported to be part of supra-molecular protein networks linking transmembrane proteins to the cytoskeleton in photoreceptor cells [11] and hair cells of the inner ear [12]. Autoantibodies to harmonin (HAA), detected by immuno-blot and radioligand assay, have been reported in IPEX patients [13] and in a small proportion of patients with colon cancer [14]. More recently, the actin-binding 95 kDa protein denominated villin, involved in the organization of actin cytoskeleton in the brush border of epithelial cells [15], was described as an additional target of autoantibodies in a proportion of patients with IPEX [16]. Conversely, to our knowledge, no information has been reported either on HAA, or villin autoantibodies (VAA) in IPEX-like syndromes, primary immunodeficiencies (PID) with enteropathy or in disorders frequently associated to IPEX, such as T1D and autoimmune enteropathies of different origin.

The aim of this study was to develop quantitative assays for the measurement of HAA and VAA based on the recently developed Luminescent Immuno Precipitation System (LIPS) [17], determine their diagnostic accuracy in the IPEX, IPEX-like and PID syndromes, evaluate their concordance with enterocyte antibodies tested by immunofluorescence, and assess their value in the clinical follow up of IPEX patients.

## Patients and Methods

### Patients and Controls

Thirteen patients with IPEX and 14 patients with IPEX-like syndrome were tested in LIPS for the presence of HAA and VAA. As control groups, we investigated 5 patients with PIDs of different origin [two with CD25 deficiency, two with Wiskott Aldrich Syndrome (WAS) and one with adenosine deaminase deficient severe combined immunodeficiency (ADA-SCID), all conditions characterized by early onset enteropathy], 123 with T1D, 70 with celiac disease and 123 healthy blood donors. IPEX diagnosis was based on clinical and molecular findings, according to the criteria defined by the Italian Association of Paediatric Haematology and Oncology (AIEOP, www.AIEOP.org). Mutations and clinical details of IPEX and IPEX-like patients are summarized in Tables S1 and S2, respectively. All IPEX patients except Pt19, Pt21, Pt22, and Pt24 were described in previous publications [3], [18]–[20]. PT24 presented with an atypical form of the disease, characterized by late onset, no signs of enteropathy, but severe gastritis in the presence of mucosal inflammatory infiltrates associated with villous atrophy. Total IgG levels were available in 10 of the 13 IPEX patients studied: of these, 8 were in the age-matched normal range (with only one patient under intravenous (IV) Ig therapy), while in two they were mildly increased. Patients diagnosed with IPEX-like syndrome had clinical manifestations of IPEX, but tested negative for mutations in *FOXP3* gene. IPEX-like patients presented at least one of the main clinical features of IPEX (autoimmune enteropathy and/or T1D) associated with one or more of the following autoimmune or immune mediated disorders: dermatitis, thyroiditis, haemolytic anemia, thrombocytopenia, nephropathy, hepatitis, alopecia, hyper IgE with or without eosinophilia. Clinical and laboratory parameters allowed exclusion of other monogenic diseases, such as WAS, Omenn’s syndrome, hyper IgE syndrome and autoimmune lymphoproliferative syndrome. At least one serum sample from patients with IPEX and IPEX-like syndromes was available for autoantibody assays at time of diagnosis. In six IPEX patients multiple serum samples were also obtained during the clinical follow up and used for additional autoantibody measurements, for studying the correlation with the clinical outcome of the disease (Pt12∶8 samples, from birth to 8 years of age; Pt14∶7 samples, from 6 months to 13 years of age; Pt17∶3 samples, from 4 months to 3.5 years of age; Pt19∶4 samples from 4 months to 2 years of age; Pt22∶3 samples between 0 and 5 months of age; Pt 23∶4 samples between 4 and 10 years of age). All patients with PIDs were diagnosed based on molecular testing. Patients with T1D were all recent onset cases, with diagnosis based on the American Diabetes Association criteria [21]; patients with celiac disease were all studied at the time of diagnosis based on jejunal biopsy.

### Ethics Statements

Written informed consent was provided by each patient and by next of kin, caretakers, or guardians on the behalf of the minors/children participants involved in this study, according to the Declaration of Helsinki. The research was approved by the San Raffaele Hospital Scientific Institute local Research Ethics Committee.

### *FOXP3* Gene Analysis

All patients classified as having either IPEX or IPEX-like syndrome were typed for *FOXP3* gene mutations. Genomic DNA was isolated from peripheral blood by using the phenol-chloroform method or the QIAamp DNA Blood Mini Kit (Qiagen), following the manufacturer’s instructions. Eleven exons, including all intron-exon boundaries, were amplified from genomic DNA by means of PCR with specific flanking intron primer pairs. The amplified gene fragments were sequenced by using the BigDye Terminator Cycle Sequencing Kit (Applied Biosystems) on an automated ABI PRISM 3130xl Genetic Analyzer and ABIPRISM 3730 Genetic Analyzer (Applied Biosystems).

### HAA and VAA Assays

The coding sequence of Renilla luciferase was cloned into the pTnT plasmid (Promega, Milan, Italy) to generate the pTnT-Rluc vector. The full length harmonin and villin DNA coding sequences were then amplified by RT-PCR and cloned separately in pTnT-Rluc downstream of and in frame with that of Renilla luciferase. Recombinant chimeric Rluc-Harmonin and Rluc-Villin were expressed by *in vitro* coupled transcription and translation using the pTnT-quick SP6 rabbit reticulocyte lysate cell free system (Promega). To test for the presence of HAA or VAA Rluc-Harmonin and Rluc-Villin were used as antigens in LIPS (17) incubating 4×10^6^ light units equivalents with 1 µl of each patient’s serum in PBS ph 7.4-Tween 0.1% (PBST) for 2 hours at r.t. IgG immune-complexes were recovered by addition of protein-A-sepharose (G.E. Healthcare, Milan, Italy) followed by 1 hr incubation at 4°C and washing with PBST of unbound Ag by filtration in Costar 3504 96-well filter plates (Corning Life Sciences, Tewksbury, USA). Immunoprecipitated antigens were then quantified by measuring the recovered luciferase activity after the addition of the Renilla luciferase substrate (Promega) and measurement of light emission for 2 sec in a Centro XS3 luminometer (Berthold Technologies GmbH & Co. KG, Bad Wildbad, Germany). Results were expressed in arbitrary units, derived either from an antibody index (VAA) using a positive and a negative serum according to the formula (cps test serum – cps negative serum)/(cps positive serum – cps negative serum)x100 or from a standard curve (HAA) consisting of serial dilutions of a positive reference serum. Cut-off for positivity was placed at the 99th percentile of the values observed in healthy blood donors, as commonly adopted in workshops for the assessment of T1D associated autoantibodies assay sensitivity and specificity [22].

### Enterocyte Autoantibody Determination

Enterocyte autoantibodies were determined in IPEX and IPEX-like patient groups by indirect immunofluorescence on cryostat sections of normal human or monkey jejunum, as previously described [7].

### T1D and Celiac Disease Specific Autoantibodies Measurement

Autoantibody markers of T1D and celiac disease, including antibodies to glutamic acid decarboxylase (GADA) [23], insulinoma-associated protein 2 [24], insulin [25], Zinc Transporter 8 [26] and transglutaminase-C [27], were measured in all IPEX, IPEX-like, PID,T1D, celiac, and healthy donor control groups by immunoprecipitation using LIPS or radiobinding as previously described. All results were expressed in arbitrary units derived from standard curves obtained by serially diluting positive reference sera.

### Statistical Analysis

Only descriptive statistics has been used in this study. Calculation of the 99^th^ percentile of arbitrary units in blood donors for threshold selection was performed using Stata (StataCorp LP, USA). The conditional probability to test positive (sensitivity) or negative (specificity) for either HAA or VAA depending on presence or absence of the IPEX disease condition and the corresponding 95% confidence intervals were calculated using the Vassar Stats website for Statistical Computation (http://vassarstats.net/clin1.html). Correlation between HAA and VAA titers was based on the Spearman rank correlation test and was calculated using the Graphpad Prism 5 software.

## Results

### HAA and VAA in IPEX, IPEX-like and Disease Control Groups

**Figure 1:**
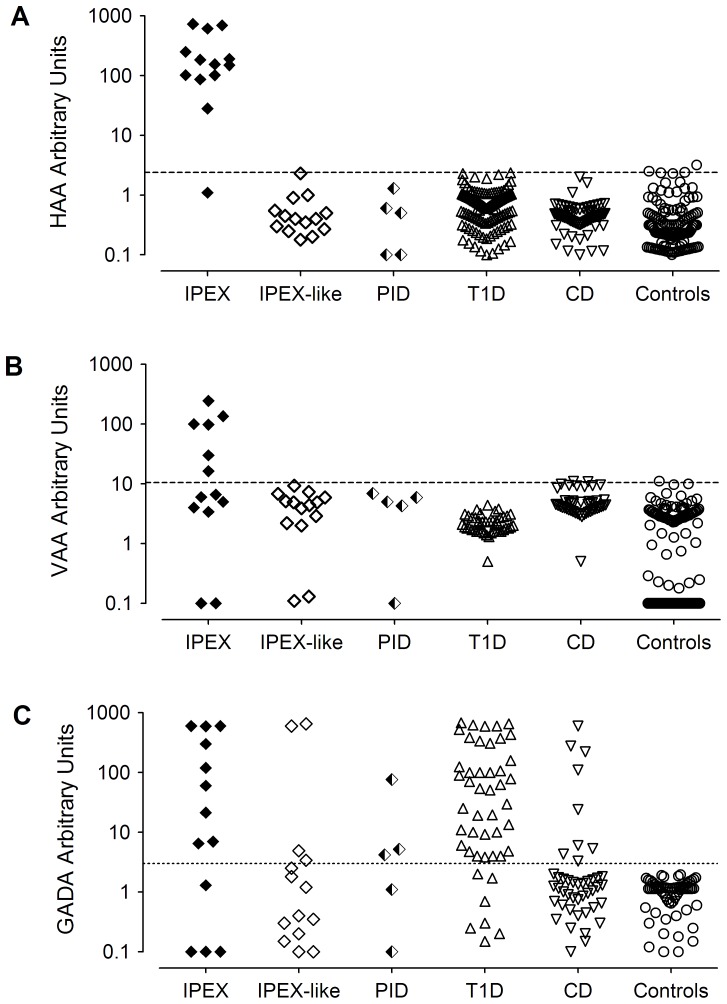
Scatter plot of HAA, VAA, and GADA titers in patients’ sera. HAA (panel A), VAA (panel B) and GADA (panel C) serum IgG titers expressed in arbitrary units in IPEX (n = 13), IPEX-like (n = 14), PID (n = 5), T1D (VAA and GADA n = 123, VAA n = 46), celiac disease patients (HAA n = 70, VAA n = 46, GADA n = 44), and in controls (HAA and VAA n = 123, GADA n = 67). Dotted line indicates the cut-off for positivity.

Elevated concentrations of circulating HAA were found in 12 of 13 (92%) patients with IPEX, while they were negative in the IPEX-like, PID, T1D and celiac disease patients (Fig. 1A). Elevated concentrations of circulating VAA were found in 6 (46%) IPEX patients (Pt19, Pt14, Pt12, Pt17, Pt3, Pt21, with the latter four having titers equal or greater than 98 VAA AU), including the patient with no HAA (Pt17), while VAA were negative in the IPEX-like and the other disease control groups (Fig. 1B). All patients with IPEX were positive for either HAA or VAA, conferring to the combination of HAA and VAA a test sensitivity of 100% (95% CI: 71.6 to 100%) and a test specificity of 97.6% (95% CI: 92.5 to 99.4%) for the diagnosis of the IPEX syndrome. No clinical or phenotypic characteristics correlated with the presence of either autoantibody in IPEX patients. No significant correlation was observed in IPEX patients between HAA and VAA autoantibody titers (Spearman r = −0.3 p = ns). GADA, as most prevalent T1D autoantibodies, were found in a proportion of patients with IPEX (9 out of 13, 5 having T1D), IPEX-like syndrome (4 out of 14, 2 having T1D) and PID (3 out 5, 1 having T1D) (Fig. 1C). Other T1D autoantibodies were found in lower proportions, including insulin autoantibodies in 5 IPEX, 4 IPEX-like and 2 PID, and Zinc Transporter 8 autoantibodies in one IPEX patient. No correlation was observed between GADA and HAA or VAA titers (Spearman r = −0.017 p = ns, and r = 0.34 p =  ns, respectively). None of the patients with IPEX, IPEX-like syndrome, or PID had celiac disease associated tissue transglutaminase-C autoantibodies of IgA or IgG class (data not shown).

### Enterocyte Antibodies

**Figure 2:**
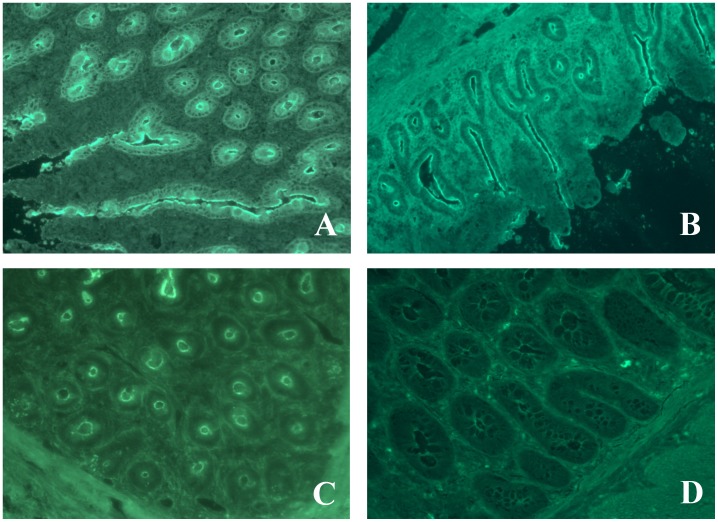
Immunofluorescent staining of intestinal enterocytes with patients’ sera. HAA from IPEX Pt 19 bind the brush border and cytosol of enterocytes (panel A) while VAA from IPEX Pt 17 binds only the brush border (panel B). IgG from PID Pt L1 bind the enterocytes brush border (panel C). Absence of binding in IPEX-like Pt L30 (panel D).

All IPEX sera but one (Pt 22), 10 IPEX-like, and 3 PID sera were tested for enterocyte antibodies by immunofluorescence on intestine cryostat sections. All tested IPEX patients were positive for enterocyte antibodies. HAA positive sera showed a strong reactivity against intestinal villi enterocytes brush border and cytosol, with highest intensity on the brush border (Fig. 2A). Isolated high titer VAA showed strong staining to brush border, but not cytosol (Fig. 2B). Outside the IPEX patient group, only one serum from a PID patient with a *CD25* gene mutation and negative for HAA and VAA (Pt L1) showed a positive staining of enterocytes limited to the brush border (Fig. 2C).

### HAA, VAA and IPEX Clinical Outcome

**Figure 3:**
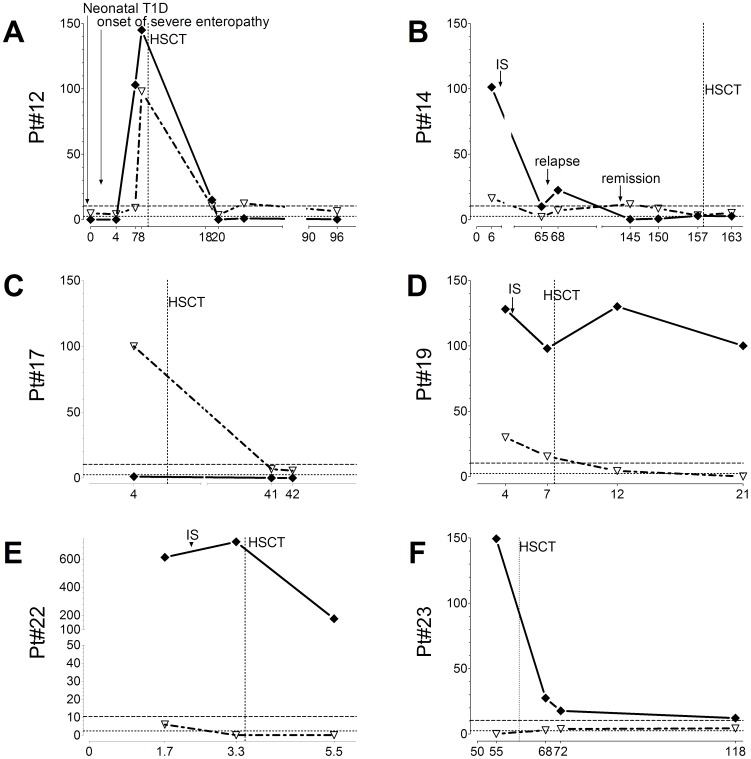
HAA and VAA titers in IPEX patients in the course of therapy. On the vertical axis are indicated HAA **(**diamonds**)** and VAA **(**triangles**)**, autoantibody titers expressed in arbitrary units, on the horizontal axis time in months. The vertical dotted line indicates the date of HSCT, horizontal dotted and dashed lines indicate the cut-off for positivity of HAA and VAA, respectively.

Follow up samples for HAA and VAA measurements were available for 6 IPEX patients (Pt12, Pt14, Pt17, Pt19, Pt22 and Pt23): all of them underwent hematopoietic stem cell transplantation (HSCT) as curative therapy, preceded in 4 cases by a variable period of systemic immunosuppression. At the time of this report (April 2013), all but two transplanted patients were alive, in clinical remission from their enteropathy, and not taking immunosuppressive therapy (Table S1). The genetic analysis of peripheral blood collected after transplant showed a 100% donor chimerism in 4 cases (Pt12, Pt14, Pt19 and Pt22), and mixed donor/recipient chimerism in the other patients. At the onset of enteropathy, three patients had both HAA and VAA (Pt12, Pt14, and Pt19), one had VAA only (Pt17) and two had HAA only (Pt22 and Pt23) (Fig. 3). In five cases (Pt12, Pt14, Pt17, Pt22 and Pt23) the clinical remission or marked improvement following either immunosuppression or HSCT was accompanied by a decrease of both HAA and/or VAA titers that became undetectable or persisted at very low titers in the four cases with the longest follow-up. In one case (Pt19), after HSCT VAA became undetectable, while HAA persisted at high titers despite clinical remission (Fig. 3D). In at least one case (Pt14), HAA proved to be a sensitive marker of enteropathy: HAA were detected at high titers in association with severe enteropathy at the time of diagnosis of IPEX, then decreased during the clinical and histological remission following immunosuppressive therapy, peaked at the time of clinical relapse, and then became persistently undetectable after successful HSCT and clinical remission (Fig. 3B). Although less prevalent, VAA showed a pattern similar to that of HAA. The drop in auto-antibodies observed after HSCT was not due to B cell and IgG deficiency secondary to conditioning. Indeed, with the exception of Pt22, who had a short follow up post transplant, all patients with decreased HAA or VAA titers after HSCT (Pt12, 17, and 23), were already immune reconstituted and IVIg therapy independent at the time of the first determination after HSCT.

## Discussion

In this study we show that HAA and VAA, easily measurable by the novel LIPS assays and used in combination, are highly sensitive and specific markers of the IPEX syndrome and may predict its clinical outcome. In fact, all IPEX patients with diagnosis confirmed by genetic testing, had elevated concentrations of either HAA or VAA. In contrast, none of the patients with enteropathy without *FOXP3* mutations (i.e. IPEX-like or PID), patients with T1D or celiac disease were positive for either HAA or VAA. Of the two markers, HAA had the highest sensitivity, being detected in 12 out of 13 patients with IPEX, while VAA were found in only six of them. Noteworthy, HAA and VAA proved to be valuable markers of IPEX syndrome also in atypical cases, such as Pt24, where enteropathy was not part of the clinical presentation, dominated instead by a severe gastritis, in whom IPEX was suspected and then confirmed by *FOXP3* gene sequencing only after the finding of elevated HAA. In the future, the new LIPS assay will allow a more systematic screening for HAA and VAA in patients with heterogeneous clinical syndromes, with the potential of identifying more cases of clinically atypical IPEX syndromes.

GADA were the second most frequent autoantibody reactivity observed in IPEX patients after HAA. Although GADA are the most prevalent autoantibody marker of T1D, with a wide titer range at the time of clinical onset [28], they are not invariably associated with diabetes. In fact, they can be found also in other autoimmune diseases, including Stiff man syndrome and autoimmune polyendocrinopathy (APS). Interestingly, in APS patients GADA are more correlated with the development of gastrointestinal symptoms rather than diabetes [29]. Intriguingly, also in our IPEX patients, GADA were largely prevalent without being invariably associated with T1D.

In addition to being accurate markers of the IPEX syndrome, HAA and VAA may have a potential predictive value, particularly with regard to the associated enteropathy. In the six patients with available follow up samples, high titers of both HAA and VAA were detected at the time of diagnosis or at the onset of gastrointestinal symptoms and prior to treatment. Afterwards, in five cases following immunosuppressive treatment and/or HSCT (Pt12, Pt14, Pt17, Pt22 and Pt23), HAA and VAA titers declined, becoming undetectable or persisting at low titers around the threshold of detection, reflecting the clinical and histological remission of the associated enteropathy. In one of them (Pt14), a transitory relapse of enteropathy occurring during the immunosuppressive treatment was accompanied by a peak of HAA, with a subsequent drop after clinical remission. Unfortunately, in this patient the lack of sequential samples prevented us from documenting the timing of the autoantibody rise preceding the enteropathy relapse. In one case (Pt19), clinical remission was accompanied by a decline of VAA, but not of HAA, that persisted at high titers up to 15 months after HSCT. The finding of a drop in HAA and VAA titers after HSCT in most, but not in all patients, is extremely intriguing, being possibly related to the survival of residual host B lymphocytes or plasmacells, responsible for the persistent production of these autoantibodies.

The introduction of these autoantibody markers in the clinical routine would be relatively simple, given the ease of their measurement by the newly developed LIPS. This technology has recently been proposed as a novel non radioactive procedure to replace the gold standard protein-A radiobinding and immunoprecipitation of in vitro transcribed and translated ^35^S-methionine-labelled recombinant human antigens, validated through established autoantibody standardization programs in both T1D [30], [31] and celiac disease [32]. In recent reports, LIPS showed performances comparable to those of radiobinding assays [33] and superior to pre-existing ELISA [34], [35]. In this study, LIPS has been developed using recombinant chimeric *Renilla* luciferase (Rluc)-Harmonin and Rluc-Villin as antigens, ensuing in assays with low background noise and linear quantitative autoantibody measurements able to discriminate positive from negative serum samples. Therefore, the measurement of HAA and VAA by LIPS proved to be a rapid, simple and reproducible test, easily applicable for clinical use.

Interestingly, the same diagnostic performance of combined HAA and VAA was observed with enterocyte autoantibodies detected by the traditional indirect immunofluorescence. It also remains unclear, but worth to test in the future, whether harmonin and villin are the only antigens recognized on enterocytes by IPEX-associated autoantibodies or if other enterocyte autoantigen targets of IPEX associated antibodies are yet to be identified.

So far, IPEX has been considered a T-cell, namely Treg-cell-dysfunction immune disease [18], [36], [37], with limited attention paid to associated defects of the humoral immune response: our findings highlight the association of the underlying *FOXP3* gene mutations with a robust and quantitatively measurable antigen-specific autoantibody response. However, since B-cells do not express *FOXP3*, *FOXP3*-mutations are unlikely to have a direct effect on B cell development and/or antibody production. Nonetheless, recent studies indicate that B cells can be both direct and indirect targets of Treg cell-mediated suppressive function [38], [39] and alteration of Treg cells affects autoantibody titers in both murine models and humans [40]–[45]. Furthermore, direct evidence from *foxp3-*mutant mice indicates that lack of Treg cells is associated with abnormal B cell development, loss of B-cell anergy and development of long-lived plasma cells [46], [47]. Moreover, it has recently been demonstrated that in humans *FOXP3* deficiency results in the accumulation of autoreactive clones in the mature naïve B cell compartment, suggesting an important role for Treg cells in the control of the peripheral B-cell tolerance checkpoint [48].

The mechanisms responsible for harmonin and villin autoimmunization in IPEX and the role of these autoantigens in the pathological manifestations of IPEX syndrome remain unknown. Harmonin is expressed in several tissues, including the small intestine, colon, kidney, eye, the inner ear vestibule and, weakly, the pancreas. In the intestine, harmonin expression is predominantly detected in the epithelial cells of the luminal surface and in the upper half of intestinal crypts [9]–[12], and is likely localized in brush border microvilli [48]; a similar localization has been shown for villin [15], [16]. Considering that the main histopatological feature of the IPEX enteropathy is villous atrophy with apoptotic cell death of enteric epithelial cells in association with moderate to marked inflammation [13], it is likely that in this context harmonin and villin might act as a relevant molecular targets of pathogenic autoimmunity.

## Conclusions

This study demonstrated that HAA and VAA measured by LIPS are accurate diagnostic markers of the IPEX syndrome, with 100% concordance with *FOXP3* gene mutations, that differentiate IPEX, including atypical cases, from other childhood disorders associated with enteropathies. Altogether, these findings indicate that HAA and VAA should be included in the diagnostic flow and clinical follow up of patients with the IPEX syndrome in whom changes in HAA and VAA titers, indicative of enteropathy relapses, may assist clinicians in making prompt therapeutic decisions.

## Supporting Information

Table S1 **Clinical features of IPEX patients.**
(XLSX)

Table S2 **Clinical features of IPEX-like patients.**
(XLSX)
